# Identification of Gentian-Related Species Based on Two-Dimensional Correlation Spectroscopy (2D-COS) Combined with Residual Neural Network (ResNet)

**DOI:** 10.3390/molecules28135000

**Published:** 2023-06-26

**Authors:** Xunxun Wu, Xintong Yang, Zhiyun Cheng, Suyun Li, Xiaokun Li, Haiyun Zhang, Yong Diao

**Affiliations:** 1 School of Biomedical Sciences, Huaqiao University, Quanzhou 362021, China; wuxunxun2015@hqu.edu.cn (X.W.);; 2 School of Medicine, Huaqiao University, Xiamen 361021, China

**Keywords:** gentian, IR, 2D-COS, ResNet, traditional Chinese medicine

## Abstract

Gentian is a traditional Chinese herb with heat-clearing, damp-drying, inflammation-alleviating and digestion-promoting effects, which is widely used in clinical practice. However, there are many species of gentian. According to the pharmacopoeia, Gentiana manshurica Kitag, Gentiana scabra Bge, Gentiana triflora Pall and Gentianarigescens Franch are included. Therefore, accurately identifying the species of gentian is important in clinical use. In recent years, with the advantages of low cost, convenience, fast analysis and high sensitivity, infrared spectroscopy (IR) has been extensively used in herbal identification. Unlike one-dimensional spectroscopy, a two-dimensional correlation spectrum (2D-COS) can improve the resolution of the spectrum and better highlight the details that are difficult to detect. In addition, the residual neural network (ResNet) is an important breakthrough in convolutional neural networks (CNNs) for significant advantages related to image recognition. Herein, we propose a new method for identifying gentian-related species using 2D-COS combined with ResNet. A total of 173 gentian samples from seven different species are collected in this study. In order to eliminate a large amount of redundant information and improve the efficiency of machine learning, the extracted feature band method was used to optimize the model. Four feature bands were selected from the infrared spectrum, namely 3500–3000 cm^−1^, 3000–2750 cm^−1^, 1750–1100 cm^−1^ and 1100–400 cm^−1^, respectively. The one-dimensional spectral data were converted into synchronous 2D-COS images, asynchronous 2D-COS images, and integrative 2D-COS images using Matlab (R2022a). The identification strategy for these three 2D-COS images was based on ResNet, which analyzes 2D-COS images based on single feature bands and full bands as well as fused feature bands. According to the results, (1) compared with the other two 2D-COS images, synchronous 2D-COS images are more suitable for the ResNet model, and (2) after extracting a single feature band 1750–1100 cm^−1^ to optimize ResNet, the model has the best convergence performance, the accuracy of training, test and external validation is 1 and the loss value is only 0.155. In summary, 2D-COS combined with ResNet is an effective and accurate method to identify gentian-related species.

## 1. Introduction

Gentian is a traditional Chinese medicine that is known to possess various pharmacological properties, including hepatoprotective, anti-inflammatory, analgesic, antitumor, stomachic, diuretic and antiviral effects [1,2,3]. Due to its rich medicinal properties, gentian has maintained a stable and long-lasting demand in the market. However, to ensure its safety and efficacy in clinical applications, there is an urgent need for the development of a fast and convenient identification method. The modernization of Chinese medicine has paved the way for advancements in the quality control of the science of Chinese medicine. One measure to ensure the safety and efficacy of Chinese medicine is to refine the proximate species of Chinese medicine and ensure the accuracy of medication. This not only promotes the development of Chinese medicine, but also contributes to the overall modernization of the field [4].

Manual identification methods, such as characterization and microscopic observation, are commonly used in the identification of gentian, but the accuracy is subjective and limited. The consequences of misclassification of gentian can be significant, making such methods unreliable. High performance liquid chromatography (HPLC), ultraviolet spectroscopy (UV) and nuclear magnetic resonance spectroscopy (NMR) are widely used [5,6,7], but they are tedious and destructive. However, infrared spectroscopy (IR) has gained popularity in recent years due to its non-destructive, convenient, and fast analysis, with high sensitivity [8]. When combined with chemometrics, it becomes an effective method for the rapid identification of herbal species, their origin and other characteristics. In our previous research, the rapid identification of gentian in different geographical locations based on IR and an improved neural network structure Double-Net was established [9]. However, IR is a single-dimensional spectrum, which may not contain sufficient information to distinguish closely related species. The improved neural network structure Double-Net used in the previous method enhanced the accuracy of the classification, but it still relied on the quality and quantity of the inputted spectral data.

To improve the resolution of the spectra and extract more information [10], the one-dimensional infrared spectrum was extended into two-dimensional correlation spectroscopy (2D-COS), which can be divided into asynchronous correlation spectra, synchronous correlation spectra and integrative spectra [11]. Two-dimensional correlation spectroscopy (2D-COS) is a dynamic spectroscopy that is induced by external perturbations. These perturbations can range from low-frequency sinusoidal disturbances to factors such as temperature, concentration, pressure, reaction time and magnetic field [12]. This novel method can provide more comprehensive spectral information of the samples and can effectively solve the problem of spectral overlap.

In the era of big data, the use of deep learning models has become increasingly popular due to the availability of powerful computing platforms and massive datasets. These models have found widespread use in various fields such as face recognition, image classification, drug discovery and speech recognition [13,14,15]. The convolutional neural network (CNN) is a deep learning architecture that uses convolutional computation and is commonly used for image classification [16,17]. It is capable of extracting local features in images through convolutional operations and can fit the structure of images more effectively than general neural networks [18]. The residual neural network (ResNet) [19] is a representative model in CNN and achieved first place in the ILSVRC 2015 image classification task competition. The problem of disappearing or exploding gradients in previous deep learning networks can be solved by ResNet, thus making the application of deep learning in image recognition more extensive.

In this study, we propose a novel approach that combines 2D-COS with ResNet to identify gentian and its closely related species. Due to the large amount of spectral data, modeling all of the data as variables can lead to increased difficulty and the introduction of noise, which can reduce the accuracy of the model. To improve the efficiency of machine learning models and avoid data redundancy, we extracted different feature bands [20] to observe whether it would enhance the model’s performance. In conclusion, the feature fusion model based on a single feature band, fusion feature bands and full band were separately analyzed and compared. By leveraging the benefits of both techniques, ResNet has advantages in identifying gentian and its closely related species.

## 2. Results

### 2.1. Data Pre-Processing

**Figure 1:**
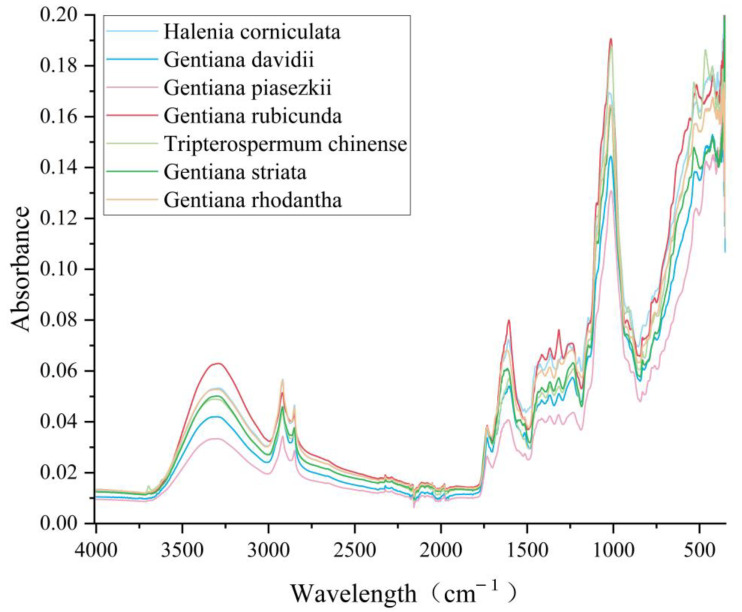
Average profiles of seven closely related gentian species.

The raw data were normalized and the average spectra were obtained using OMNIC 9.77 (Thermo Fisher Scientific, Waltham, MA, USA). The full band average spectra of seven gentian relatives are presented in Figure 1. Although the peak locations were generally similar, there were differences in peak intensities.

### 2.2. Results of Feature Variable Selection

The infrared spectra exhibited a diverse composition with characteristic bands for various substances, such as esters, saccharides, lignin, etc. Firstly, the selection of feature bands was based on peak positions while also considering factors such as peak shape and discriminability; secondly, feature bands that contained prominent peak positions were correlated with the absorption characteristics of different substances. For example, during the band selection, we noticed the characteristic peak position 3292 cm^−1^ in the 3500–3000 cm^−1^ range, as well as the characteristic peak positions 2922 cm^−1^ and 2849^−1^ in the 3000–2750 cm^−1^ range. The feature peak at 2922 cm^−1^ corresponds to the methylene asymmetrical stretching vibration. The feature peak at 2849 cm^−1^ corresponds to the methylene symmetrical stretching vibration. Other characteristic peak position groups and their vibration types are shown in Table 1. These characteristic peak positions are crucial for distinguishing different substances. Additionally, we strived to maintain the richness of information within the selected bands to enhance the model’s recognition accuracy. Therefore, we avoided selecting excessively narrow band ranges. Lastly, we also took into account the spatial relationship between feature peak positions. When closely spaced feature peaks were present, we grouped them within the same band, such as grouping 2922 cm^−1^ and 2849 cm^−1^ in the 3000–2750 cm^−1^ range. The ResNet model can comprehend and leverage the spatial relationship and mutual information between features, thereby improving its performance.

Based on the analysis of characteristic peak locations, taking into account factors such as peak shape and discriminability, four characteristic bands were selected, namely, 3500–3000 cm^−1^, 3000–2750 cm^−1^, 1750–1100 cm^−1^ and 1100–400 cm^−1^. We separately analyzed and compared the feature fusion model based on a single feature band, fusion feature bands and full band.

### 2.3. Two-Dimensional Correlation Spectroscopy (2D-COS) Image Acquisition

**Figure 2:**
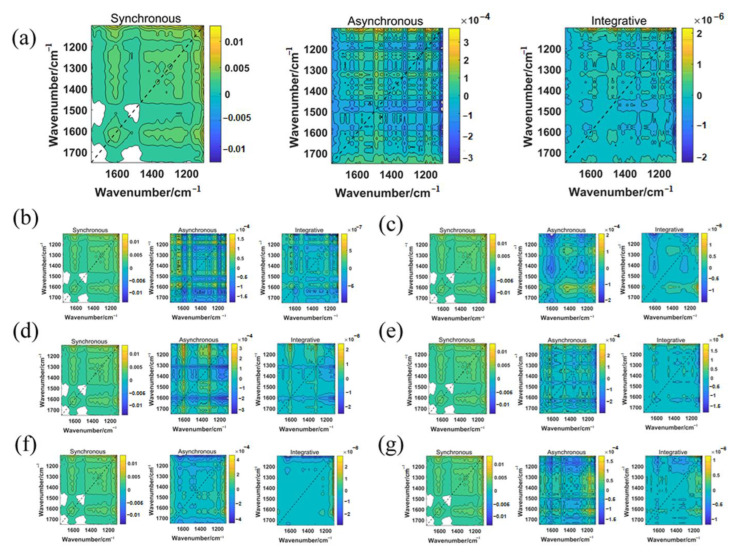
Average two-dimensional spectrum of seven closely related Gentian species based on 1750–1100 cm^−1^ bands (from left to right: synchronous, asynchronous and integrative two-dimensional spectra, respectively): (**a**) *Gentiana rhodantha*; (**b**) *Halenia corniculate*; (**c**) *Gentiana piasezkii*; (**d**) *Gentiana rubicunda*; (**e**) *Tripterospermum chinense*; (**f**) *Gentiana striata*; and (**g**) *Gentiana davidii*.

According to the results of the feature variable selection, 10% of the samples were initially selected for external validation, and the remaining model set was split into training and test sets using the Kennard–Stone algorithm, with 70% as the training set and 20% as the test set. One-dimensional spectral data were converted into synchronous 2D-COS images, integrated 2D-COS images and asynchronous 2D-COS images using Matlab (R2022a). Figure 2 and Figure S1 display the 2D-COS images obtained from the average spectral data of seven gentian species in the 1750–1100 cm^−1^ band.

### 2.4. Discrimination Results of ResNet

**Figure 3:**
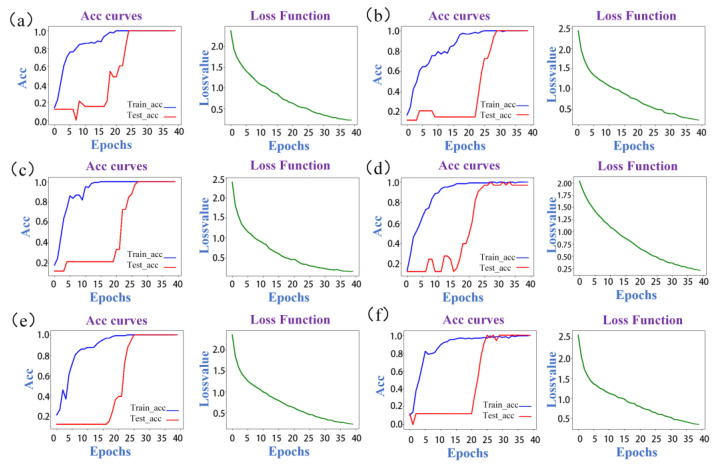
The accuracy curves and cross-entropy cost function of synchronous 2D-COS. (**a**): 3500–3000 cm^−1^; (**b**): 3000–2750 cm^−1^; (**c**): 1750–1100 cm^−1^; (**d**): 1100–400 cm^−1^; (**e**): fusion feature bands; (**f**): full band.

The modeling results of ResNet are presented in Table 2. Based on the results of previous experiments, we found that synchronous 2D-COS images are more effective in discriminating between gentian species compared to asynchronous and integrative 2D-COS images. This could be attributed to synchronous 2D-COS images having clearer line information and more characteristic peaks in the self-peaks and cross-peaks compared to the other two spectra. Therefore, they were better suited for identifying gentian species. Consequently, only synchronous 2D-COS images for subsequent analysis were selected. The accuracy curves and cross-entropy cost function of the ResNet model based on synchronous 2D-COS images from different bands are shown in Figure 3. The *x*-axis represents the number of epochs, while the *y*-axis represents the accuracy of the training and test sets. A higher value of accuracy closer to 1 indicated a stronger and more accurate model. The accuracy curves demonstrated a similar increasing trend for both the test and training sets, indicating the model’s stability and absence of overfitting. The cross-entropy loss function was used to assess the model’s convergence, with a lower value indicating better performance. Moreover, external validation was employed to prevent overfitting, and the accuracy of the validation set was compared to that of the test set. The similarity between the accuracy of the test and validation sets indicated the reliability and stability of the model. The results showed that the models based on the synchronous 2D-COS images generated from the band 1750–1100 cm^−1^ had the highest accuracy across all of the sets when epoch = 40, with a loss value of 0.155, indicating better performance.

By comparing the results of the feature band fusion models, it can be observed that the fusion of multiple bands does not improve the model’s performance, which suggests that the spectral feature information may not be proportional to the model’s discriminative ability. On the other hand, the use of a single feature band may produce a more concise model that excludes redundant information while retaining the necessary recognition features. Therefore, the single band approach may be advantageous over the feature band fusion approach.

We can conclude the following: (1): Synchronous 2D-COS images are more effective in identifying gentian species due to clearer line information and characteristic peaks. (2): The ResNet model using synchronous 2D-COS images from the band 1750–1100 cm^−1^ achieved the highest accuracy, indicating better performance. (3): The fusion of multiple bands did not improve the model’s performance, suggesting the advantage of a single feature band approach in excluding redundant information while retaining necessary recognition features.

## 3. Discussion

Previous studies have emphasized the importance of developing a fast and convenient identification method for gentian due to its rich medicinal properties and stable market demand [21,22]. However, manual identification methods, such as characterization and microscopic observation, suffer from subjectivity and limited accuracy. To address these limitations, our study introduces IR for gentian identification.

Infrared spectroscopy offers advantages such as non-destructive analysis, rapidity, high sensitivity, informativeness and programmability [23,24]. However, several limitations still exist. It may struggle to process or extract effective information when dealing with complex samples or high-dimensional data, resulting in insufficient accuracy.

In previous studies, IR has been used for the identification of gentian. For instance, Wu et al. effectively differentiated gentian samples from different geographical origins using the PLS-DA model combined with infrared spectroscopy, which could not be determined accurately as they were in the margin or outside of the 95% confidence ellipses [25]. Additionally, Shen et al. integrated infrared spectroscopy with feature selection and stacked generalization, identifying the SG-Ven-MIR-SVM model as the optimal method for discriminating closely related species of gentian [26]. Herein, our proposed method exhibits significant advantages compared to traditional approaches. By transforming the data into image format and utilizing ResNet for identification, we provide richer visual features and more detailed data representation. Additionally, considering the spatial relationships between features and leveraging the deep learning capabilities of ResNet, subtle differences among samples can be better captured and classification accuracy can be improved. Compared to digit-based models, image-based methods offer higher robustness and generalization, enabling better adaptation to variations and unknown scenarios. Furthermore, our method requires minimal data pre-processing, further enhancing its practicality and efficiency. Therefore, our approach demonstrates remarkable advantages in terms of identification capability, data processing and classification tasks.

To address these limitations, we propose the integration of the two-dimensional correlation spectrum (2D-COS) and residual neural network (ResNet) into IR analysis. By extending the one-dimensional IR spectra, 2D-COS was able to extract more detailed information and enhance the spectral resolution. Furthermore, the integration of ResNet, a powerful deep learning technique, mitigates the challenges associated with complex data and enhances the classification capabilities of the identification process. ResNet’s ability to handle deep networks and its residual connections enable the effective learning and extraction of features from the 2D-COS images of gentian species. By leveraging ResNet’s strengths, our approach addresses the limitations of traditional IR analysis and enhances the accuracy and reliability of gentian identification.

Nevertheless, it is important to acknowledge that while our proposed method shows promising results, there are still areas for improvement. One limitation of our study is the relatively small size of the dataset used for validation. The limited sample size could impact the generalization and robustness of our model. By increasing the dataset’s size and diversity, we can improve the reliability and accuracy of our proposed method. Additionally, a larger dataset would allow for more comprehensive evaluation and validation of the model’s performance.

Furthermore, the effectiveness of our approach can be further enhanced by exploring additional feature extraction methods. Different feature bands and their combinations can be investigated to determine the optimal configuration for gentian identification. By incorporating a wider range of spectral features, we can improve the discriminative power of our model and enhance its ability to differentiate between different gentian species.

In conclusion, our study demonstrates that the ResNet combined with the 2D-COS approach is a highly accurate and effective method for identifying gentian-related species. Our results show that the single-feature-band-based model outperforms both the full spectra and the feature band fusion models in optimizing the model. Moreover, this method requires less complex data pre-processing than traditional machine learning approaches like SVM and RF [27], making it a promising technique for use in the fields of food and medicine. This method not only expands the application field of deep learning but also promotes the development of drug identification and analysis technology in a more efficient and intelligent direction. It is a viable option for the rapid identification of gentian-related species and offers insights into the differentiation of other traditional Chinese medicines and agricultural products. Our findings provide a novel approach to the identification problem, wherein data can be transformed into images for use in deep learning networks, taking advantage of the image recognition capabilities of deep learning. However, this approach requires expertise in the corresponding dataset for effective utilization, as relying solely on dimensionality reduction may not yield optimal results [28].

## 4. Materials and Methods

### 4.1. Sample Information

In this study, 173 gentian samples were collected from seven different species. All samples were identified by Associate Professor Xianxiang Xu of the College of Medicine at Huaqiao University. Fresh gentian was washed with deionized water, and then treated by being placed in an electric blast dryer and dried at a constant temperature of 60 °C. The resulting dried powder samples were crushed using a high-speed crusher and sieved through a 100 mesh sieve. The processed gentian was stored in sealed polyethylene bags under dry conditions, awaiting further processing.

### 4.2. IR Acquisition

The FT-NIR spectrometer used in this study was the Thermo Fisher Nicolet iS 50. Scanning was conducted within the range of 4010–350 cm^−1^, with a resolution of 4 cm^−1^, and the signal was scanned 64 times. Each sample was scanned three times to obtain the average value. Due to excessive noise, the range of 365–350 cm^−1^ was excluded, and the 4010–365 cm^−1^ band was used for subsequent analysis. The resulting spectra were converted to datasets using SIMCA-P+14.1 (Umetrics, Umea, Sweden) [10].

### 4.3. Two-Dimensional Correlation Spectroscopy (2D-COS) and Integrated 2D-COS Spectra Image Acquisition

#### 4.3.1. Introduction of 2D-COS

The generation of two-dimensional correlation spectra relies on changes in spectral intensity caused by the external perturbation variable *t*, resulting in dynamic spectra $\bar{y}\left(v,t\right)$ [12].

The dynamic spectrum $\bar{y}\left(v,t\right)$ is defined as follows:

(1) $$\bar{y}\left(v,t\right)=\left\{\begin{array}{c} y\left(v,t\right)-\bar{y}\left(v\right)\;\;\;\;\;\;\;T_{min}\leq t\leq T_{max}\\ 0\;\;\;\;\;\;\;\;\;\;\;\;\;\;\;\;\;\;\;\;\;\;\;\;\;\;\;\;\;other \end{array}\right.$$

In practical experiments, the dynamic spectrum is in discrete form and the dynamic spectral intensities are expressed as a column vector *y* of variable *v* when the spectra with an equal interval of perturbation t are measured in *n* steps:

(2) $$y\left(v\right)=\left\{\begin{array}{c} y\left(v,t_{1}\right)\\ y\left(v,t_{2}\right)\\ \vdots \\ y\left(v,t_{n}\right) \end{array}\right\}$$

Φ is the synchronous two-dimensional correlation intensity, which can be expressed as follows:

(3) $$\Phi \left(v_{1},v_{2}\right)=\frac{1}{n-1}y{\left(v_{1}\right)}^{T}.y\left(v_{2}\right)$$

ψ is the asynchronous two-dimensional correlation intensity, which can be expressed as follows:

(4) $$\psi (v_{1},v_{2})=\frac{1}{n-1}y{\left(v_{1}\right)}^{T}.N.y\left(v_{2}\right)$$

*N* is the Hilbert–Noda matrix, and is defined as follows:

(5) $$N_{jk}=\left\{\begin{array}{c} \;\;0\;\;\;\;\;\;\;\;\;\;\;\;\;\;\;\;\;\;\;j=k\;\\ \frac{1}{\pi \left(k-j\right)}\;\;\;\;\;\;j\neq k \end{array}\right.$$

*I* is the asynchronous two-dimensional correlation intensity, which can be expressed as follows:

(6) $$I=\psi \left(v_{1},v_{2}\right).\Phi \left(v_{1},v_{2}\right)$$

#### 4.3.2. The 2D-COS Algorithm

In this study, the following steps were involved in the 2D-COS algorithm:

Firstly, we introduced external perturbations to the spectral data by applying a sinusoidal perturbation. This perturbation, in the form of a sinusoidal wave, introduced variations in the spectral intensity at specific spectral points, allowing us to analyze the dynamic changes in the spectral data [29].

Next, a set of dynamic spectral data was obtained based on the application of these perturbations. These perturbed spectra were then organized into a matrix format. Subsequently, we performed mathematical correlation analysis on the matrix to identify the interrelationships between different spectral components. This analysis involved the calculation of synchronous and asynchronous as well as integrative correlation spectra.

Finally, 2D-COS were generated as graphical representations. These correlation spectra provide visual insights into the dynamic behavior and interdependencies of the spectral features.

By following these steps, incorporating the sinusoidal perturbations, we were able to effectively analyze the spectral data and generate two-dimensional correlation spectra, which offer valuable information for understanding the underlying dynamics and relationships within the spectral data.

**Figure 4:**
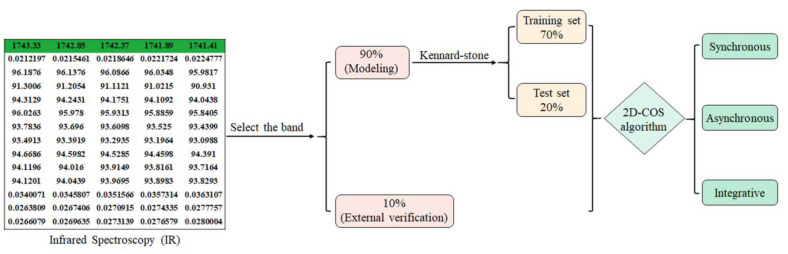
The 2D-COS image generation process.

**Figure 5:**
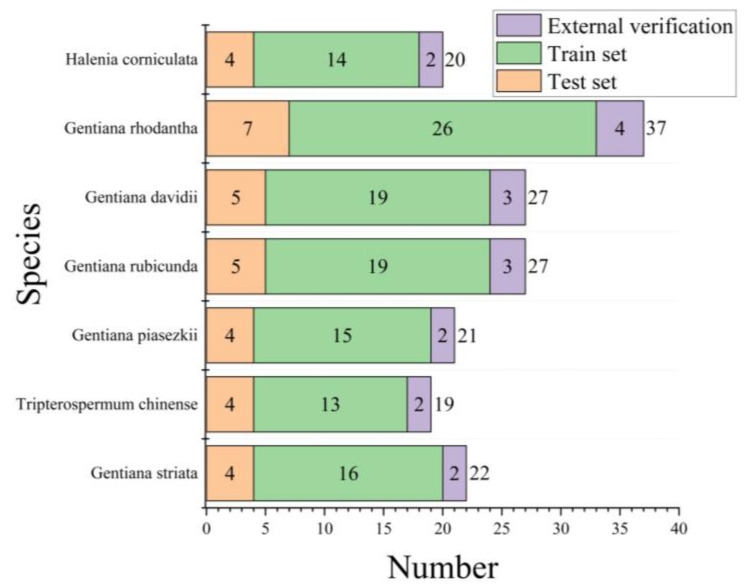
Number of training, test and external validation sets for seven gentian species.

Using Matlab (R2022a), three 2D-COS images were generated, and the generation process is shown in Figure 4. The number of sets for the seven gentian-related species is presented in Figure 5.

### 4.4. CNN

The use of convolutional neural networks (CNNs) has become increasingly popular in the field of computer vision due to their high performance in tasks such as image classification and face recognition [30]. The mechanism behind CNN involves the feature extraction of input data through convolutional kernels, also referred to as filters. These filters are passed over the image in a specific step and padding configuration, with the process being repeated until the entire image is covered [31,32].

The formula to calculate the dimensions of the output matrix is as follows, *n_out_*, where *p* is the padding, *f* is the filter dimension, *s* is the stride and *n_in_* is the input image:

(7) $$n_{out}=\frac{n_{in}+2p-f}{s}+1$$

**Figure 6:**
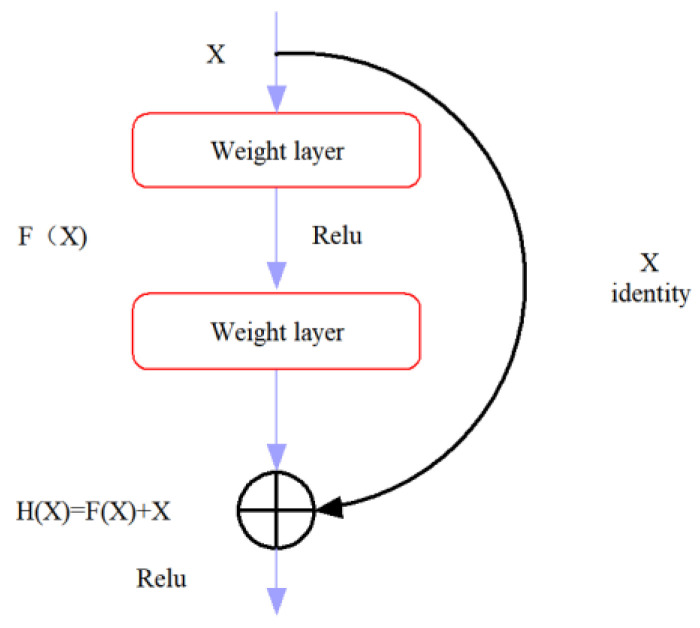
Structure diagram of the ResNet model residual blocks.

**Figure 7:**
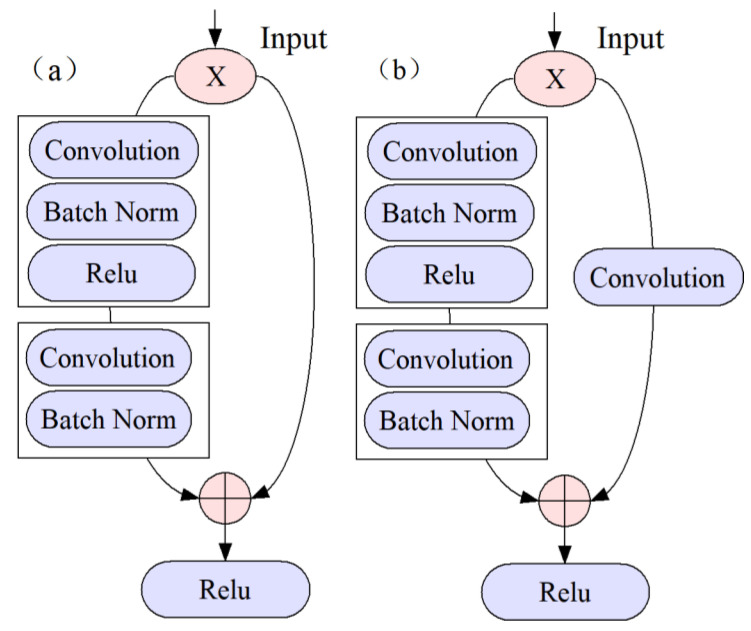
(**a**): Identity residual block; (**b**): convolution residual block.

**Figure 8:**
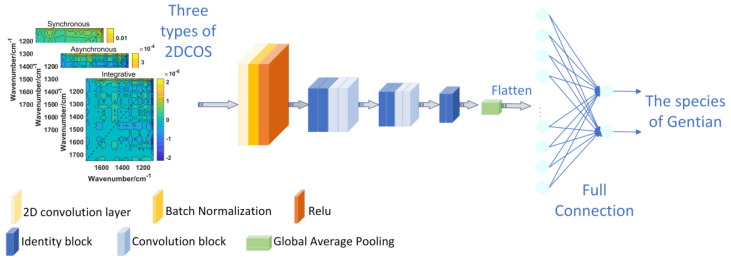
The ResNet model that was used in the study.

CNN is a deep learning architecture in which VGGNet (visual geometry group network) [33] and GoogLeNet [34] have achieved impressive performance by increasing the number of layers in the network. However, some problems such as vanishing or exploding gradients can arise by increasing the number of layers in deep learning, which can cause the training and testing error rates to increase. These issues are not related to overfitting and must be carefully addressed during model development [35].The introduction of ResNet has significantly improved this challenge. ResNet is designed to preserve information integrity and simplify the learning objectives and difficulty through the use of skip connections with residual mapping [36] Additionally, ResNet is able to increase accuracy through increased depth [37]. In ResNet, the internal residual modules utilize shortcut connections, which allow the direct transfer of the input X to the output. This mechanism ensures the integrity of information throughout the network. The output result is computed as H(x) = F(x) + X, where x represents the shallow output, and H(x) represents the deep output. Consequently, the objective function is reformulated as F(x) = H(x) − x, and the residual block structure of the ResNet model [19] is depicted in Figure 6. The residual block can be classified into two categories based on the dimensions of the input X and the output F(x). If they have the same number of dimensions, the identity block is used, as shown in Figure 7a. Alternatively, if they have different dimensions, the convolution block is used, as shown in Figure 7b. Based on these two types of residual blocks, ResNet was constructed, with the model structure depicted in Figure 8.

By incorporating ResNet into our approach, we were able to leverage its ability to handle deeper networks and address the issues related to gradient vanishing or exploding. This enabled us to effectively learn and extract features from the 2D-COS images of gentian species, resulting in improved accuracy in the identification process. The integration of ResNet’s residual connections allowed for the direct flow of information, preventing the degradation or loss of information as the network depth increased. This design choice played a crucial role in our model’s performance, enabling us to effectively capture the intricate patterns and details present in the 2D-COS images of different gentian species.

During the model building process, the learning rate was set to 0.01 and the weight attenuation coefficient was 0.0001. Firstly, the 2D-COS images of synchronous, asynchronous and integrative two-dimensional spectra were inputted, respectively. A convolution layer was applied to the input data, and BatchNorm was used to normalize the data, which effectively prevents overfitting. Subsequently, a non-saturated non-linear activation function ReLu was applied to the data, and the output was fed into six identification blocks and four convolution blocks to extract features. Then, global average pooling was performed for each feature map to reduce the number of parameters, effectively avoiding overfitting and converting the data into one-dimensional data. After all the convolution and pooling operations, the obtained feature maps were flattened by rows and concatenated into vectors, which were then fed into the full connection layer.

**Figure 9:**
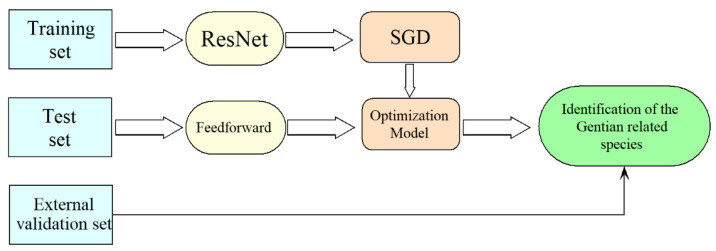
Identification strategy for gentian closely related species based on ResNet.

The identification strategy for closely related gentian species based on ResNet is shown in Figure 9. To enhance the model’s performance, data augmentation was performed, followed by weight update and cross-entropy loss reduction using the stochastic gradient descent (SGD) method [38]. The optimal model was obtained when the iteration number or error reached the threshold. The trained neural network was then utilized to classify the images in the test set via forward propagation (feedforward). Finally, the model’s generalization ability was assessed using an external validation set.

## 5. Conclusions

In this study, we successfully combined computer science with Chinese medicine to develop an effective method for identifying gentian-related species using ResNet combined with 2D-COS. Our study not only contributes to the identification of gentian species, but also provides insights into the identification of other Chinese medicines.

Although the sample size for validation was limited, we were able to achieve promising results on the available dataset. We acknowledge the limitations of our sample size and emphasize the need for future studies to include a larger sample set to further validate and enhance the reliability of our proposed method.

In addition, we suggest the following directions for future research: (1) Future studies can focus on extending ResNet combined with 2D-COS to higher-quality images and optimizing the model parameters to improve the accuracy of identification. (2) In addition to ResNet, other models such as CNN with ViT (vision transformer) and DenseNet (dense convolutional network) can be explored to classify two-dimensional spectral images. (3) Fine-tuning can be used to optimize the model training process, which involves specific pre-processing and dropout tuning to adapt to different types of datasets.

## Figures and Tables

**Table 1:** Different characteristic peak position groups and their vibration types.

Feature Bands (cm^−1^)	Wavenumber (cm^−1^)	Vibration Mode
3500–3000	3292	O-H stretching vibration
3000–2750	2922	Methylene asymmetrical stretching vibration
	2849	Methylene symmetrical stretching vibration
1750–1100	1732	Ester substance C=O stretching vibration
	1610	Terpenoid C-C asymmetrical stretching vibration
	1510, 1422	Lignin benzene ring skeleton vibration
	1371	Methylene deformation vibration
1100–400	1030	Saccharide C-OH stretching vibration
	920	C-H bending vibration

**Table 2:** The ResNet modeling results. (Syn-: synchronous correlation spectrum; Asy-: asynchronous correlation spectrum; Int-: integrative spectrum.)

Model Number	Band (cm^−1^)	The Type of 2D-COS	Loss Value	Train Set Acc	Test Set Acc	Validation Set Acc
A	3500–3000	Syn-	0.222	100.00%	100.00%	100.00%
		Asy-	1.277	59.84%	36.36%	44.40%
		Int-	1.187	67.21%	30.30%	50.00%
B	3000–2750	Syn-	0.227	100.00%	100.00%	100.00%
		Asy-	1.483	68.85%	36.36%	27.78%
		Int-	1.31	64.75%	45.45%	44.44%
C	1750–1100	Syn-	0.155	100.00%	100.00%	100.00%
		Asy-	1.346	67.21%	54.55%	38.89%
		Int-	1.153	63.93%	63.64%	66.67%
D	1100–400	Syn-	0.202	100.00%	96.97%	100.00%
		Asy-	1.304	63.11%	45.45%	27.78%
		Int-	1.265	59.02%	51.52%	44.44%
E	A + B + C + D	Syn-	0.223	100.00%	100.00%	100.00%
F	Full spectra	Syn-	0.339	100.00%	100.00%	100.00%

## Data Availability

The data presented in this study are available on request from the corresponding author.

## Supplementary Materials

The following supporting information can be downloaded at: https://www.mdpi.com/article/10.3390/molecules28135000/s1, Figure S1: Average two-dimensional spectrum of seven closely related dragon gall species based on bands 1750–1100cm^−1^ (from left to right, synchronous, asynchronous, and integrative two-dimensional spectra, respectively) (b) *Halenia corniculate*; (c) *Gentiana piasezkii*; (d) *Gentiana rubicunda*; (e) *Tripterospermum chinense*; (f) *Gentiana striata*; (g) *Gentiana davidii*.

## Sample Availability

Samples of the compounds are available from the authors.
