# Supplementary material for: Identification of Gentian-Related Species Based on Two-Dimensional Correlation Spectroscopy (2D-COS) Combined with Residual Neural Network (ResNet)
Source: Molecules. 2023 Jun 26;28(13):5000. doi: 10.3390/molecules28135000 (PMC10343857; doi:10.3390/molecules28135000)
Supplement: Supplementary file 1 [file molecules-28-05000-s001.zip › molecules-2432901-supplementary.pdf]

Identification of Gentian-Related Species Based on Two-Dimensional Correlation Spectroscopy (2D-COS) Combined with Residual Neural Network (ResNet)

Xunxun Wu [1], Xintong Yang [1], Zhiyun Cheng [1], Suyun Li [2], Xiaokun Li [1], Haiyun Zhang [2] and Yong Diao [1],*

1 School of Biomedical Sciences, Huaqiao University, Quanzhou 362021 , China;

wuxunxun2015@hqu.edu.cn (X.W.); yangxintong@stu.hqu.edu.cn (X.Y.);

chengzhiyun@hqu.edu.cn (Z.C.); lixiaokun@stu.hqu.edu.cn (X.L.)

2 School of Medicine, Huaqiao University, Xiamen 361021, China; lisuyun@hqu.edu.cn (S.L.); haiyunzhang@stu.hqu.edu.cn (H.Z.)

*Correspondence: diaoyong@hqu.edu.cn

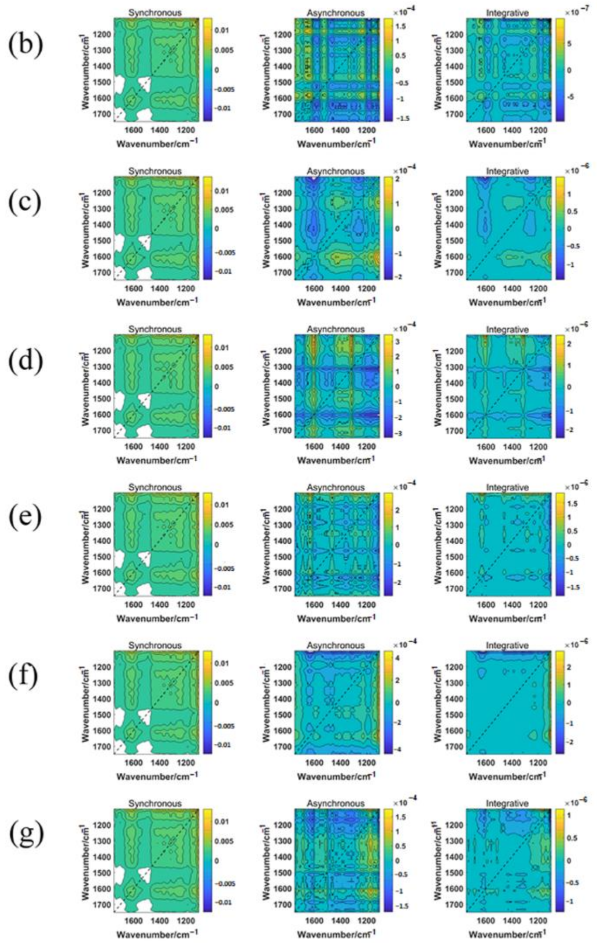

Figure S1. Average two-dimensional spectrum of seven closely related gentian species based on bands 1750-1100cm^{-1}(from left to right, synchronous, asynchronous, and integrative two-dimensional spectra, respectively) (b) Halenia corniculate; (c) Gentiana piasezkii; (d) Gentiana rubicunda; (e) Tripterospermum chinense; (f) Gentiana striata; (g) Gentiana davidii.
